# Towards a Conceptual Participatory Framework to Promote Health Literacy in Adolescents by Integrating Self-Determination Theory and Game Design

**DOI:** 10.3390/ijerph23030328

**Published:** 2026-03-06

**Authors:** Michela Franchini, Giada Anastasi, Stefania Pieroni, Francesca Denoth, Benedetta Ferrante, Alessia Formica, Sabrina Molinaro

**Affiliations:** 1 Institute of Clinical Physiology, National Research Council, 56124 Pisa, Italy; giadaanastasi@cnr.it (G.A.); francesca.denoth@cnr.it (F.D.); benedettaferrante@cnr.it (B.F.); alessiaformica@cnr.it (A.F.); sabrina.molinaro@cnr.it (S.M.); 2 Department of Molecular Biology, University of Siena, 53100 Siena, Italy

**Keywords:** adolescents, health literacy, serious games, self-determination theory, Octalysis, Dress-DIGITARIAN

## Abstract

**Highlights:**

**Public health relevance—How does this work relate to a public health issue?**

- High exposure to online content combined with limited critical skills can contribute to mental health problems, unhealthy body image, risky behaviours, and reduced self-esteem among adolescents, with consequences that can persist into adulthood.
- Health literacy is a key factor in disease prevention and behavioural change.

**Public health significance—Why is this work of significance to public health?**

- The use of a theory-driven serious game provides a scalable, engaging, and prevention-oriented intervention that can be implemented in school settings, aligning with population-level health promotion strategies.
- The One Digital Health (ODH) framework, which integrates the One Health approach by leveraging digital technologies to collect, process, and share data, information, and knowledge across multiple health-related sectors, provides a meaningful contribution to advancing adolescent mental health promotion and modernizing public health strategies in digital environments.

**Public health implications—What are the key implications or messages for practitioners, policy makers and/or researchers in public health?**

- The development of policies that promote inter- and transdisciplinary thinking across different sectors of society more effectively address complex health challenges.
- Adolescent’s involvement in decision-making roles ensures that research reflects their unique needs and perspectives and empowers young people as agents of change, thereby increasing the relevance and impact of adolescent health promotion.

**Abstract:**

Adolescents are heavy users of digital media but often lack critical skills, increasing their vulnerability to harmful online content. The integration of game elements into learning and training offers a promising strategy to support positive behavioural change and strengthen adolescents’ skills. This paper describes the development of a conceptual framework for Dress-DIGITARIAN, a serious game aimed at improving health literacy, coping skills, and self-esteem, grounded in Self-Determination Theory (SDT). The framework was constructed to generate higher-order understanding through a multi-level process: analyzing general theory (SDT), integrating mid-range models (the Octalysis framework), and incorporating empirical insights derived from two data collection phases with the target population. This integrative approach informed and guided the game’s design through participatory methods. Developed through collaboration between schools and research institutions, this approach bridges theory and practice by aligning game mechanics with adolescents’ psychological needs. It also underscores the value of involving adolescents in research, not only to enhance scientific rigour but also to empower them as agents of change capable of contributing to health promotion policies and educational innovation. This study does not report the results of a completed intervention or outcome evaluation, which will be conducted in the sixth phase at the end of the current school year. Future research is needed to assess the model’s effectiveness and scalability and to identify areas for further refinement.

## 1. Introduction

Health literacy is an individual-based construct acquired in a life-long learning process, starting in early childhood. It has a multidimensional nature, interacting in different contexts (e.g., education, sport, family) and settings (e.g., school, afterschool, daily life, and public health) [1].

Theoretical and operational knowledge are fundamental aspects of health literacy. Knowledge acquisition depends on the individual’s ability to access information and comprehend it. The ability to comprehend health information also implies the ability to filter and extract only relevant information and to appraise it. Critical thinking is crucial particularly from adolescents’ perspective, because it requires a complex evaluation of (a) accuracy, validity, and appropriateness (correct information or the message’s credibility); (b) impartiality (unbiased communication); (c) relevance (applicability to the problem); (d) comprehensiveness (broad coverage of the information); and (e) internal consistency (logical relationships between information and/or concepts) [2,3].

Currently, digital revolution is increasingly influencing health, and the impact of individual digitals skills on health literacy seems to be greater than formal education levels. Numerous international studies indicate that young people have the highest digital media usage rates (e.g., Instagram, Snapchat, YouTube, TikTok) across all age groups, dedicating a substantial amount of their time to these platforms [4,5].

The European School Survey Project on Alcohol and Other Drugs (ESPAD) providing comparable data on substance use and risk behaviours among secondary school students has been conducted every four years, since 1995, in 37 European countries. In Italy, nearly all ESPAD students (93%) use digital devices. Problematic Internet Use (PIU) between 2021 and 2024 affected 13% of students 15–19 years of age, with 49.8% of them spending more than 6 h online during a school day. When daily internet use exceeds four hours, a higher prevalence is observed among girls [6,7].

Students’ primary interests often revolve around topics like body transformations, diet/nutritional supplements or recipes and workouts/exercises (#thinspo and #fitspo messages). These habits and interests have led to adult concerns about the spread of misinformation and disinformation that may promote harmful health practices, including eating disorders or overly restrictive diets [8].

Despite their high levels of digital competence and extensive use of social media, public discourse frequently represents young people as deficient in critical skills, lacking the ability to differentiate between sources, analytically interpret meaning and relevance, and apply information to personal health, thus constructing them as especially vulnerable to the harms of online content. Consequently, research on adolescent and digital media has traditionally focused on the negative consequences of its use. More recently, the growing popularity of AI language-based chatbots such as ChatGPT has enhanced the debate on the risks of providing inappropriate or harmful safety-related information [9].

This narrative of young people as actors passively exposed to online health-related information reduces the potential for new technologies to be used for developing innovative and more effective health promotion interventions [10,11].

Conversely, positive media psychology focuses on the positive emotional, cognitive and behavioural effects of technology engagement in supporting wellbeing [12,13]. A growing body of research explores gamification—the integration of game elements into learning and training—to examine its potential in motivating behavioural change [14]. Existing studies have frequently overlooked strategies for selecting game elements and for integrating behaviour change theories, either separately or in combination, within the game design process. A recent review identified the Self-Determination Theory (SDT) as the most cited and adopted theoretical framework in behaviour change games studies, in particular for strengthening autonomous decision-making and promoting analytical and critical thinking among players [15,16].

The SDT states that the primary function of the self during development is to assimilate, regulate, and coordinate inputs from both external sources—socio-cultural environment—and internal sources—drives and emotions—and proposes that for individuals to grow and thrive, they need to satisfy three fundamental needs in the social context: perceived competence, autonomy, and relatedness [17].

When individuals feel autonomous, competent, and accepted, they experience high self-determination, leading to enhanced self-motivation and wellbeing across various fields of life, such as education [18], sport and physical activity [19,20], religion [21] and healthcare [22].

According to a recent research [23], the SDT could provide a valid framework to develop more sophisticated and flexible strategies in gamification design by (a) incorporating diverse and dynamic goals in gamification following both controlled-motivation and autonomous-motivation approaches; (b) addressing multiple psychological concurrent needs and evaluating how meeting one need influences others and (c) recognizing the influence of individual differences and contextual factors to develop personalized and effective systems.

Building on these theoretical foundations, we aim to co-design, in collaboration with high school students, a serious game entitled Dress-DIGITARIAN—DIGItal Technology for heAlth pRomotIon And educatioN of young people. The tool belongs to the DRESS system (Doing Risk sElf-assessment and Social health Support), a family of easy-to-use tools developed to promote a proactive approach to health in the general population through a collaborative information pathway [24]. The Dress-DIGITARIAN is thought to promote adolescents’ health literacy and foster practical self-management skills across the primary domains that shape adolescents’ online information-seeking behaviours—nutrition, physical activity, and self-image—while simultaneously promoting coping abilities and the development of self-esteem.

To engage adolescents in the co-design process, we are conducting a participatory action research (PAR) project in a general high school setting, within the framework of the Pathways for Transversal Skills and Orientation (PCTO), an educational programme aimed at students enrolled in the last three high school years in Italy [25,26]. The research project integrates game-based learning principles with the SDT to cultivate intrinsic motivation and sustained engagement among participating students and their peers.

This paper describes the development of a conceptual framework for Dress-DIGITARIAN along a six-step process. It includes a synthesis of the existing literature across multiple theoretical perspectives (general and midrange theories) to form novel, higher-order understanding by integrating knowledge from the empirical domain (two data collections within the target population) to structure and guide the tool design though a participatory approach. The proposed framework aims to ensure that every component—from the narrative structure to the feedback system—highlights the most influential game elements and mechanics adopted to achieve the greatest impact from the adolescents’ perspective.

## 2. Materials and Methods

We adopted the Intervention Mapping (IM) approach, as a practical bridge between theory, research evidence, and real-world practice, ensuring that the game framework is both effective and contextually appropriate [27]. The IM is a systematic planning framework that draws upon theoretical models and empirical evidence to design, implement, and evaluate health interventions. A key principle of IM is the active engagement and participation of target communities from problem assessment and needs analysis to programme development, implementation, and evaluation. Additionally, we referred to the World Health Organization (WHO) framework guiding the development and implementation of youth-focused digital health interventions [28]. The framework highlights the importance of meaningful youth engagement throughout all phases—from conceptualization and design to delivery, monitoring, and evaluation. By involving young people as active participants rather than passive recipients, the framework promotes interventions that are more relevant, user-friendly, and impactful.

Therefore, we adopted a PAR approach that emphasizes collaboration and shared decision-making among an interdisciplinary group of researchers (epidemiologists, kinesiologists, nutritionists, psychologists and IT scientists), and secondary school students acting as co-researchers [25].

**Figure 1:**
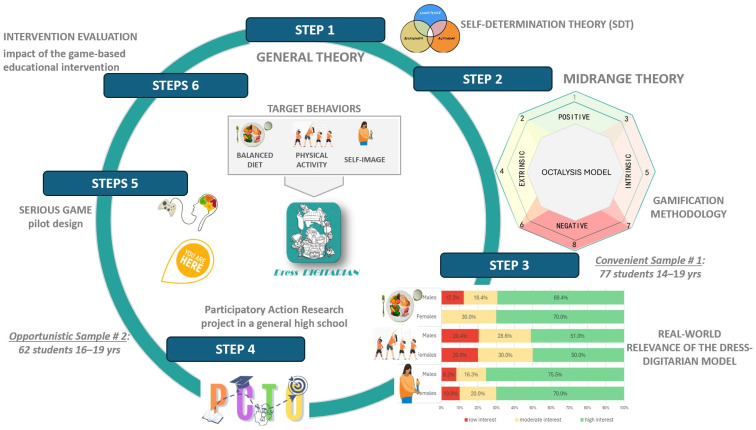
Dress-DIGITARIAN research protocol.

This approach emphasizes participation, engagement, empowerment, mutual learning, capacity building, and the pursuit of aligned research and action goals. Grounded in the core SDT principles of autonomy, competence, and relatedness, this approach is intended to support and strengthen adolescents’ wellbeing. Our primary aim is to foster co-researchers’ critical thinking by empowering them to effect positive change in their own lives and in the lives of their peers. Inspired by the D6 Gamification Framework [29] and its subsequent refinements, our research protocol follows six key steps: (a) from a scientific standpoint, understanding the needs of the target population, identifying the target behaviours to be promoted and the psychological approach to identity formation (general theories); (b) mapping the needs to suitable gamification design strategies (midrange theory) (c) assessing adolescents’ interests to drive the PCTO design; (d) involving PCTO students in theoretical lectures and interactive, game-based activities; (e) developing and iteratively refining a pilot design of the game; and (f) formulating a pilot evaluation plan that encompasses both effect and process evaluations (Figure 1).

The first two steps articulate the theoretical framework, whereas the third and fourth steps delineate the conceptual model of the Dress-DIGITARIAN.

Because the third and fourth steps involve human participants, including individuals under the age of 18, the study protocol has been approved by the Commission for Research Ethics and Bioethics of the National Research Council in Italy on 11 April 2019 (protocol N. 0027159/2019). Moreover, all parents of participating students provided written informed consent for the project’s activities and for the anonymous use of collected data to be published in aggregate form.

In the third step, a sample of 77 high school students aged 14 to 19 years (Figure 1) were engaged in a brief anonymous web survey to (a) evaluate their interest in self-image, (b) determine their desire to gain knowledge about balanced nutrition and physical activity, (c) collect their suggestions on additional topics of interest and need and (d) evaluate their competence about balanced nutrition, nutritional supplements and the effects of physical activity.

Participants were recruited using a non-probability convenience sampling method. The survey was made accessible via a QR code to all students at a secondary school, and participation was entirely voluntary. As a result, the sample was self-selected. Demographic information such as age class (14–15 years; 16–19 years) and gender was collected to monitor the representativeness of the sample.

Based on the evidence from the first three steps, a 6-month action-research programme was designed within a school setting (PCTO) to advance the development of the fourth and fifth steps of the research protocol.

At present, a second cohort of 62 students aged 16 to 19 years belonging to three distinct classes of a general high school in Italy is participating in the PCTO (Figure 1). It comprises a series of activities focused on nutrition, physical activity, self-image, and game design theory. The purpose of these activities, led by the interdisciplinary project group of researchers, is to equip students with foundational competence that enables their active engagement in the Dress-DIGITARIAN co-design process. The pilot design of the Dress-DIGITARIAN represents the final output of the PCTO project (step 5).

The guidelines developed to support the effective implementation of PCTO emphasize the importance of systematically documenting each learning experience and disseminating outcomes among students and teachers to encourage the sharing of best practices. In accordance with these recommendations, two anonymous web-based questionnaires were administered to the participating students before the project began. The first questionnaire (FQ) comprised 27 items designed to assess students’ baseline competencies across the relevant domains regarding the functioning of the nervous system and emotional processes, physical activity, nutrition, and the responsible use of social media and digital technologies. The second questionnaire (SQ), consisting of 33 items, was designed to evaluate students’ well-being, and perceived self-efficacy in managing positive and negative emotions [30,31,32]. This approach is supported by research showing that individuals who are confident in managing negative emotions can reduce depression and delinquency while enhancing life satisfaction. Similarly, confidence in expressing positive emotions is associated with higher self-esteem, optimism, prosocial behaviour, emotional stability, happiness, and overall daily contentment [31,32]. Specifically, the SQ included the Italian adaptation of the 18-item version of the Ryff’s Psychological Well-Being Scale [30], 7 items from the APEP/G scale and 8 items from the APEN/G scale to evaluate the perceived ability to regulate one’s own positive (e.g., joy, love, and amusement) and negative (e.g., anger, sadness, and fear) emotions, respectively [31,32]. All three scales demonstrated good test–retest reliability, with Cronbach’s alpha higher than 0.8 [30,31,32]. The Ryff’s scale evaluates six dimensions: self-acceptance (SA), defined as a positive attitude toward oneself and one’s past; positive relations with others (PRs), defined as the ability to have a satisfying relationship with others, autonomy (AU), reflecting independence and self-determination, environmental mastery (EM), defined as the perceived competence to manage one’s environment and external demands, purpose in life (PL), defined as having goals for the future and a sense of direction, and personal growth (PG), defined as the positive attitude towards one’s development [30]. The 18-item version of Ryff’s Psychological Well-Being Scale is available in Appendix B of Sirigatti et al., 2009 [30].

The FQ and SQ will be re-administered at the conclusion of the project to examine longitudinal changes and to determine the effects of scientific information and game-based learning on students’ health literacy and psychological well-being (Step 6). 

## 3. Theoretical Framework

### 3.1. Step1: General Theories

Numerous definitions of adolescents’ wellbeing have been proposed, with two main primary conceptual approaches: subjective or internal wellbeing and objective or external wellbeing [33].

In the context of identity formation, eudaimonic well-being is a crucial element highly recognized by the SDT as well. Discovering the true self means uncovering personal potentials, identifying one’s life purposes and seeking opportunities to express and fulfil those potentials and purposes [34].

A large body of evidence shows that entering high school is a notably stressful time: the shift from primary to secondary school involves a major change in environment, including the loss of familiar peer groups and the support of primary school teachers. At the same time, students face heightened academic and social pressures, requiring them to adapt to a new setting and navigate the expectations associated with this stage of growth. The most common stressors contributing to lower subjective well-being include academic struggles, social challenges, and mental health issues [35]. This change and transition in many aspects of life may exceed adolescents’ coping capacity and increase the risk of developing a distorted self-image and impair self-esteem [36].

Self-image is a multidimensional construct, significantly influencing adolescents’ identity formation and impacting life satisfaction, depression, and anxiety. Self-image is strictly related to nutritional habits and physical activity [37,38,39].

The balance between psychological need satisfaction and frustration is critical, as adolescents who experience chronic dissatisfaction with their self-image may engage in maladaptive coping strategies, such as social withdrawal, perfectionism, eating disorders or compulsive behaviours [40,41,42].

According to the DSM-5, the prevalence of eating disorders among children and adolescents (aged 11–19 years) ranges from 1.2% in boys to 5.7% in girls, with incidence increasing steadily over recent decades. However, these figures are likely underestimated due to substantial rates of underdiagnosis and undertreatment. A recent meta-analysis conducted by López-Gil and colleagues [43], which included 32 studies with large samples from 16 countries, reported that more than one in five children and adolescents exhibit disordered eating—defined as a spectrum of problematic eating patterns and attitudes toward food, body weight, shape, and appearance that are known to predict eating disorder diagnoses in early adulthood. The study also found that disordered eating is more prevalent among females and among young people with excess body weight compared to their normal-weight peers [43].

Moreover, negative coping strategies as well as depression, anxiety, and low self-esteem play a strong predictive role in the development of mental health problems and eating disorders [44].

The Italian COPE-NVI version categorizes coping into five primary strategies: seeking social support, avoiding stressors, facilitating problem-solving, positively reframing stressful events and their outcomes, and adopting a transcendent approach through reflection, philosophy, or spirituality [45].

A recent study by Zammuner V.L. [46] confirmed that avoidance is a form of maladaptive coping, while the other strategies are positively associated with all wellbeing indicators, but the association tends to vary between strategies. Gaming, by presenting players with various virtual scenarios through the gaming narrative, can promote health literacy to indirectly strengthen adolescents coping skills and encourage strategies that go beyond mere avoidance [40]. Therefore, the game approach can integrate scientifically validated information to equip players with new knowledge and skills to enhance competence, autonomy and resilience [47]. Furthermore, by challenging the distorted belief of permanence—the idea that negative emotions or situations will persist indefinitely—it aims to increase players’ ability to recover from different adversities or challengers [48].

Integrating game narratives with challenges that promote self-improvement—such as enhancing self-image (e.g., guiding players through experiences that counter negative self-talk and strengthen self-efficacy), encouraging balanced nutrition (e.g., designing tasks centred on healthy meal choices that yield in-game rewards or avatar enhancements), and fostering physical activity (e.g., implementing progressive exercises, such as stretching or workouts, that gradually increase in difficulty)—provides the game system with clear direction and purpose.

Furthermore, because game feedback mechanisms can either facilitate or hinder behavioural change, actively involving students in a co-design process enables the presentation of scientific evidence in an age-appropriate and engaging manner to peers, while fostering practical knowledge in self-managing their own health [49,50,51]. When embedded within a gamified learning environment, feedback provides opportunities for iterative experimentation, reflection, and immediate reinforcement, supporting motivation, self-efficacy, and the internalization of adaptive behaviours. Aligning feedback strategies with gamification principles and the psychological needs outlined in the SDT further enhances students’ engagement and facilitates the translation of theoretical knowledge into practical, transferable skills. This design approach supports meaningful cognitive and behavioural change among players [49].

### 3.2. Step 2: Midrange Theory Linking SDT to Gaming Design

The game system’s structure consists of the mechanisms and actions that can influence or modify the framework. Gamification mechanics are the specific rules, systems, and interactions (e.g., points, badges, levels, leaderboards, rewards) driving engagement, retention, and gaming behaviour. To define the Dress-DIGITARIAN mechanic, we refer to the Octalysis Framework, developed by [50]. It is a human-focused gamification design model that reorganizes the main mechanics of gamification into eight Core Drives: (1) meaning, (2) development and accomplishment, (3) empowerment of creativity and feedback, (4) ownership and possession, (5) social influence and relatedness, (6) scarcity and impatience, (7) unpredictability and curiosity and (8) loss and avoidance (Figure 1).

**Figure 2:**
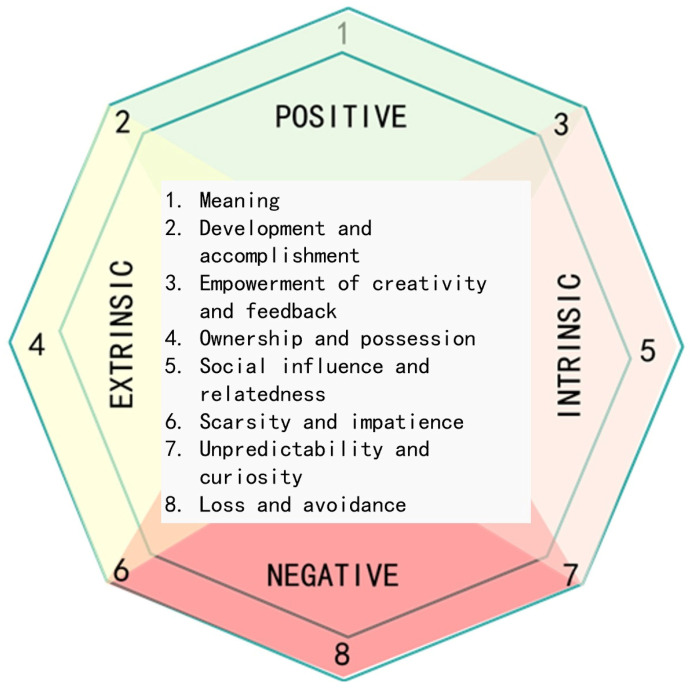
Octalysis Framework Model [50].

Octalysis distinguishes between Left Brain Core Drives (vertices 2, 4 and 6, reflecting logic, calculations and ownership), identified as extrinsic motivators, and Right Brain Core Drives (vertices 3, 5 and 7, linked to creativity, self-expression, social aspects), which are intrinsic motivators, more aligned to the SDT (Figure 2).

Octalysis also differentiates between positive motivators (White Hat Core Drives: vertices 1, 2, 3) and negative motivators (Black Hat Core Drives: vertices 6, 7, 8). While positive motivators create a more satisfying gaming experience, techniques that rely on negative motivators may leave gamers feeling manipulated [50].

During the development or evaluation of a gamification design, the Core Drives are assigned an individual score between 0 and 10 to provide quantitative and qualitative feedback for each game goal (Table 1). Therefore, the higher the score, the more pronounced the Core Drive and the more used a game strategy.

Furthermore, the Octalysis Framework aims to create a gaming experience, keeping players engaged at every stage of their journey (discovery, onboarding, scaffolding and endgame) by satisfying various Core Drives at different stages [50].

**Figure 3:**
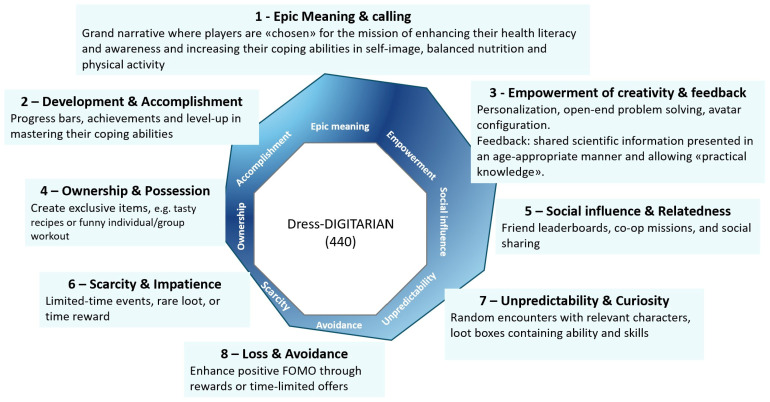
General view of Dress-DIGITARIAN Core Drives [50].

In light of the above, and following the Octalysis framework (Figure 2), we designed the Dress-DIGITARIAN mechanic with a stronger focus on White Hat (vertices 1, 2 and 3) and Right Brain Core Drives (vertices 3, 5 and 7), aligning with the SDT principles and intrinsic motivation (Figure 3).

## 4. Towards the Conceptual Model

### 4.1. Step 3: Empirical Knowledge Domain

To explore the real-world relevance of the proposed game scopes, a brief anonymous web survey was administered to a convenience sample of 77 high school students from 14 to 19 years of age (33.8% female; 95% CI: 24.2–44.9%; 62.3% aged 16–19 years; 95% CI: 51.2–72.3%).

Data were collected during a designated week of educational activities. The QR code to access the survey was placed in multiple locations in the school to ensure voluntary participation and protect participant anonymity.

Consequently, the sample was self-selected, with females underrepresented relative to the national distribution of Italian high school students (49.9% female; 95% CI: 49.8–49.9%).

The survey aimed to explore and describe students’ interest in and awareness of key health domains, including nutrition (“acquiring new knowledge about nutritional needs during adolescence and nutritional balance”), physical activity (“acquiring new knowledge about the value of physical activity in promoting health”), and self-image (“promoting awareness and appreciation of one’s self-image”), using the prompt “How relevant are these objectives to you?” (Figure 1). Additionally, the survey invited students to propose objectives they personally considered important, capturing needs not previously identified from a scientific perspective. Finally, it assessed students’ interest in participating in the Dress-DIGITARIAN project by asking, “Would you like your high school to launch a PCTO based on the Dress-DIGITARIAN project?”.

**Figure 4:**
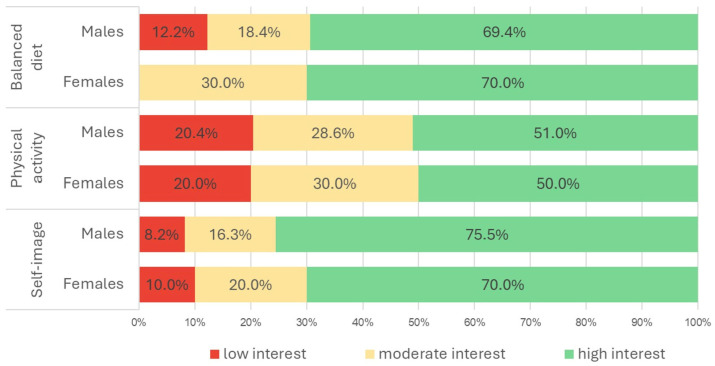
Students’ interest in the paradigm and goals of the Dress-DIGITARIAN.

Notably, 73.9% (95% CI 69.3–82.5%) of the students expressed a strong interest in self-image issues (Males: 75.5%; Females: 70.0%). Additionally, 69.6% (95% CI 59.1–79.2%) indicated a strong interest in balanced nutrition (Males: 69.4%; Females: 70.0%), while half of the respondents (50.7%; 95% CI: 39.7–61.5%) showed interest in acquiring knowledge about physical activity (Figure 4).

**Figure 5:**
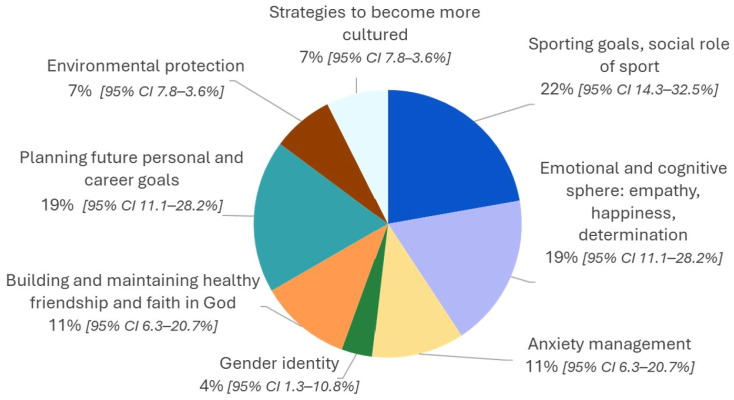
Students’ suggestions on additional topics to be faced by the Dress-DIGITARIAN.

Additionally, most students suggested exploring topics related to their psychological and emotional well-being, including empathy, happiness, determination (19%; 95% CI: 11.1–28.2%), and anxiety (11%; 95% CI: 6.3–20.7%). Specifically, 11% (95% CI: 6.3–20.7%) of adolescents suggested focusing on strategies to enhance relatedness, such as fostering and maintaining healthy friendships and emphasizing the role of religion as a coping mechanism. Notably, 22% (95% CI: 14.3–32.5%) of respondents—predominantly males—showed interest in promoting the role of physical activity and sports in personal and social development, supporting autonomy and relatedness. Finally, consistent with the need for competence, nearly one in five students (19%; 95% CI: 11.1–28.2%) expressed interest in developing strategies for planning their personal and career goals (Figure 5).

**Figure 6:**
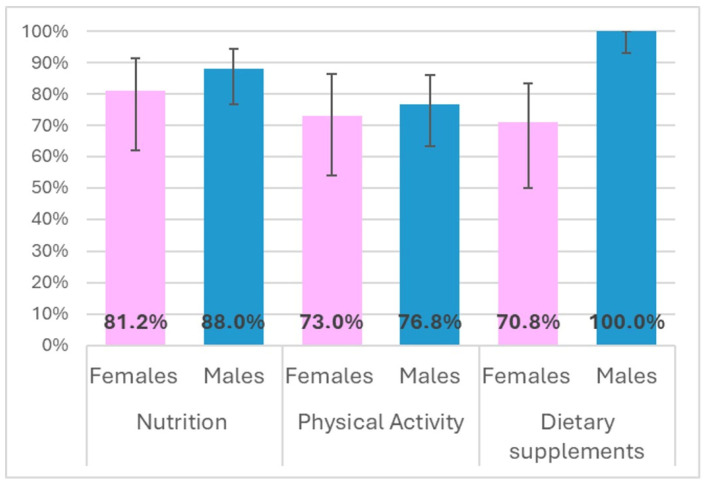
Percentage and variability (95% CI) of correct answers about balanced nutrition, nutritional supplements and the effects of physical activity.

Students were also asked to choose the right answer to questions assessing their knowledge of nutrients and nutrition (9 items), the healthy use of fitness and dietary supplements (6 items), and the effects of physical activity and sports (9 items). The results indicated that students’ knowledge of nutrition and the use of supplements was fragmented, particularly among female participants (Figure 6). Moreover, knowledge about the effects of physical activity was the most limited (correct answers: 75.6%; 95% CI: 64.6–83.6% vs. 85.7% nutrition and 90.2% supplementation), reflecting a generally low level of fitness-related motivation [52].

Finally, 95.7% (95% CI: 89.2–98.7%) of the surveyed students (Males: 95.9%; Females: 95.0%) indicated that they would like their high school to implement a PCTO inspired by the Dress-DIGITARIAN project.

### 4.2. Step 4: The PCTO Design and Ongoing Results

An opportunistic sample of 62 students (3 school classes) attending the third and fourth (out of five) years of upper secondary education at a science-focused high school in Italy (aged 16–19 years; females 41.9%;95% CI: 30.5–54.3%) participated in the project through teacher involvement (Figure 1). Compared to the first group of surveyed students, this sample is more closely aligned with the sex distribution of Italian high school students (females 49.9%; 95% CI: 49.8–49.9%).

A four-session instructional program was implemented to equip students with foundational scientific knowledge in nutrition, physical activity, self-awareness, responsible internet and social media use, and basic game theory. Each session combined a focused theoretical lecture with interactive, game-based activities that employed purpose-built pedagogical toolkits—e.g., wooden food models for nutrient categorization and dietary planning aligned with the Harvard Healthy Eating Plate framework. Sessions also incorporated physical workouts of varying intensity to foster students’ autonomy in self-regulating activity levels, as well as logic-based challenges designed to promote the responsible use of artificial intelligence (AI). In performing PCTO activities, students will be guided to interact critically with AI language-based chatbots, applying knowledge acquired across the three domains to strengthen their analytical reasoning, evaluative judgement, and capacity for knowledge integration.

To date, students are collaborating to produce an output including: the game’s narrative (Core Drive 1, Figure 2), strategies for goal attainment (Core Drive 2, Figure 2), challenges, structural feedback and progression dynamics (Core Drives 3, 7, Figure 2), and social sharing (Core Drive 5, Figure 2). Each class is working on a single key domain (self-image, nutrition, or physical activity). The deadline for sharing the outputs is set for May 2026. Students will present their work to the research group in a scientific format, thereby enhancing their ability to communicate research findings effectively.

As the project also aimed to evaluate the impact of the gaming-based educational intervention on students’ health literacy and psychological well-being, (Step 6), two questionnaires were administered at the beginning of the project to establish a baseline, which will be compared with a second measurement collected at the end of the school year (Figure 1). The first questionnaire (FQ) assessed students’ competencies across key domains, including the functioning of the nervous system and emotional processes, physical activity, nutrition, and the responsible use of social media and digital technologies.

**Figure 7:**
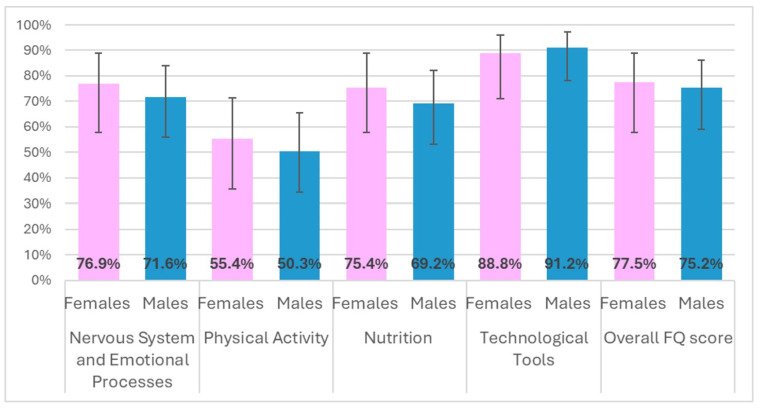
Percentage and variability (95% CI) of correct answers to the FQ items.

Baseline responses to the FQ indicated that males and females reported comparable percentages of correct answers (Males: 75.2%; Females:77.5%, Chi-square test, *p* = 0.8). Moreover, data showed that knowledge regarding the effects of physical activity (percentage of correct answers: 52.4; 95% CI: 39.4–63.6%) was lower than knowledge of technological tools (90.2%;95% CI: 84.4–95.5%), particularly among males (Figure 7).

The second questionnaire (SQ) was administered to explore students’ well-being and perceived self-efficacy in managing positive and negative emotions through three psychometric validated scales.

**Figure 8:**
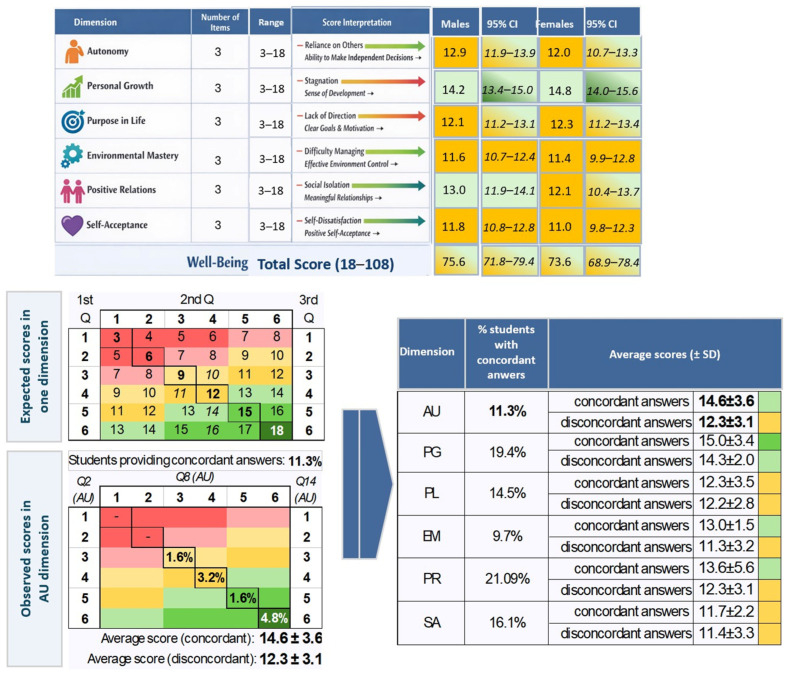
Average scores on Ryff’s scale.

The 18-item version of Ryff’s Psychological Well-Being Scale [30] assesses six dimensions: self-acceptance, positive relations with others, autonomy, environmental mastery, purpose in life, and personal growth. Each dimension is measured using three items, rated on a six-point Likert scale. Of the 18 items, six are reverse-scored using the formula (number of scale points + 1) − respondent’s answer. Consequently, scores for each dimension range from 3 to 18, while the total score for the full scale ranges from 18 to 108 (Figure 8).

To categorize wellbeing levels in each dimension, we referred to the expected score matrix (Figure 8), considering responses 1–2 for each question (dimension score from 3 to 6, indicated in the figure by dark red color) as indicative of poorer wellbeing, 3–4 as moderate (dimension score from 9 to 12, indicated in the figure by dark yellow color), and 5–6 as reflecting the best condition (dimension score from 15 to 18, indicated in the figure by dark green color). Color gradients spanning the red, yellow, and green spectrum represent wellbeing levels on a continuous scale. Consequently, in each dimension, we computed the average score by sex. Results showed general moderate-to-high psychological well-being (all average values from 11.0 to 14.8), with no statistically significant differences between dimensions and sexes.

We also computed the average scores by considering the degree of accordance among the three questions of each dimension. Students providing concordant answers compared to others did not show different levels of wellbeing (Figure 8).

**Figure 9:**
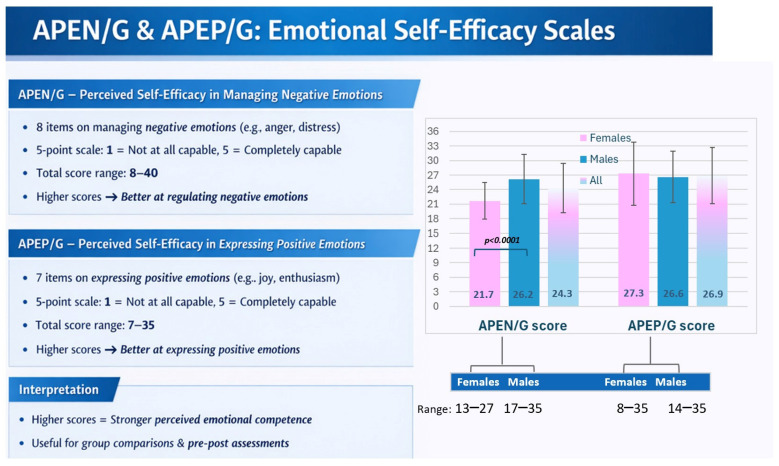
Average scores and variability (±SD) on APEP/G and APEN/G scales.

The SQ also incorporated seven items from the APEP/G scale and eight items from the APEN/G scale to assess participants’ perceived ability to regulate positive and negative emotions, respectively (Figure 9). The APEP/G scores range from 8 to 35 among females and from 14 to 35 among males, where score 8 indicates the worst and score 40 indicates the highest level of ability in managing positive emotions. The estimated average values showed no significant sex differences (Males: 26.6 ± 5.3; Females: 27.3 ± 6.5; Student’s *t*-test, *p* = 0.6). In contrast, the average value of APEN/G scores (Figure 9) revealed a statistically significant sex difference (*p* < 0.001), with females reporting a lower perceived ability to regulate negative emotions (21.7 ± 3.8) compared to males (26.2 ± 5.1).

## 5. Discussion

Health promotion focuses on identifying and leveraging individual, community, and environmental assets—such as skills, social networks, and supportive settings—to enhance population health. It also acts as a powerful catalyst for inter- and transdisciplinary thinking, fostering new networked governance approaches such as Healthy Cities and Healthy Schools [53]. Developing healthy behaviours and promoting good health among adolescents is a complex public health challenge that necessitates a comprehensive understanding of target populations, alongside the implementation of innovative, theoretically grounded strategies [54,55].

This work presents the Dress-DIGITARIAN project, which is designed to develop a serious game aimed at enhancing adolescents’ health literacy and strengthening practical self-management skills in areas that shape their online information-seeking behaviours—nutrition, physical activity, and self-image. By incorporating game-based mechanics, the Dress-DIGITARIAN project seeks to enhance adolescents’ competencies, coping abilities, and self-esteem in accordance with the SDT principles of competence, autonomy, and relatedness.

In this study, we outline a six-step process that progresses from a theoretical framework—encompassing models of adolescent self-improvement and psychological well-being, as well as established gaming theories—to the development of the Dress-DIGITARIAN design (Figure 1).

Consistently with the United Nations’ strategy, which recommends including adolescents in decision-making roles to ensure that research reflects their unique needs and perspectives, we involved two groups of Italian students [56]. The first group (*n* = 77 students) was engaged to explore and describe students’ interests across the proposed domains. Given the complex and dynamic nature of adolescent engagement, the second sample (*n* = 62 students belonging to three different classes) was selected by their teachers to participate in a PCTO project, ensuring sustained and active involvement. After completing two baseline questionnaires assessing their knowledge and psychological well-being, students participated in ongoing PCTO activities that combine structured theoretical instruction with interactive, game-based learning tasks. The PCTO aims to provide students with scientific knowledge about nutrition, physical activity, self-awareness, responsible use of the internet and social media, and basic game theory, thereby enabling them to actively contribute to the conceptualization and design of the Dress-DIGITARIAN game, which represents the project output (Figure 1).

A post-intervention assessment, designed to evaluate the impact of the game-based educational intervention on students’ health literacy and psychological well-being, will be conducted at the end of the school year through a second round of data collection (Step 6). Based on these results, our research group will refine the serious game design to produce the final game specifications; e.g., since females reported a lower perceived ability to regulate negative emotions, the game narrative and goal attainment strategies will be differentiated by sex.

The project aligns with the One Digital Health (ODH) framework, which integrates the One Health approach by leveraging digital technologies to collect, process, and share data, information, and knowledge across multiple health-related sectors [57,58].

It is also consistent with the Horizon Europe Work Programme 2026–2027, which supports the design of interventions that foster healthy habits and lasting behaviour change among youth [59], as well as with the 2030 Agenda for Sustainable Development, which encourages the active involvement of adolescents in health research [60].

Furthermore, by leveraging collaboration between schools and research institutions, the project promotes a constructivist approach to education based on active knowledge construction, experience-based learning, and collaborative activities.

## 6. Implications, Limitations, and Future Directions

The current theoretical understanding of the psychological mechanisms underlying gamification, particularly within the context of serious games and game-based learning, remains fragmented [49]. While existing research consistently demonstrates that game-based interventions can promote behaviour change, limited knowledge about the underlying processes persists, especially concerning the specific design components that influence engagement and effectiveness [15].

Furthermore, a recent bibliometric analysis revealed a significant increase in studies on gamification in health education between 2018 and 2024. However, the top 10 most cited studies primarily focus on the effectiveness of gamification in medical education [61].

The Dress-DIGITARIAN project participates in addressing new evidence on the potential of gamification in the field of health promotion among adolescents. The secondary goal is to offer serious game designers a new view towards personalized gamification experiences for use in health promotion fields. Recognizing that one-size-fits-all approaches to gamification often fail to account for individual differences in motivation, engagement, and behaviour, with the risk of promoting over engagement, the Dress-DIGITARIAN projects seeks to offer a theoretically grounded, data-driven framework for tailoring game elements to meet diverse user needs.

By integrating scientific evidence into the design of personalized gamified experiences, the project aims to bridge the gap between research and real-world application, ensuring that gamification in health promotion is not only engaging and immersive but also scientifically validated and effective. It is particularly important for promoting physical activity and fostering relatedness, with a focus on increasing off-screen activities rather than online interactions.

Finally, by leveraging the PCTO framework as a local example of a collaborative learning model bridging schools and research institutions, and by actively engaging adolescents as co-researchers, our project underscores the transformative potential of young people as agents of change.

This study has several limitations that should be considered when interpreting the results and in guiding future research. First, the empirical component is based on limited and opportunistic samples of students, which restrict the data analysis to exploratory and descriptive purposes only. Second, the study is heavily focused on Italian students and on the general education track. Future research should include more numerous samples of student from additional educational pathways (e.g., vocational and technical schools) and consider other collaborative learning frameworks beyond the Italian PCTO, as implemented in different countries. Third, this work does not report the results of a completed intervention or outcome evaluation, which will be conducted in the sixth step at the end of the current school year.

## 7. Conclusions

This work offers an example of a scalable, youth-centred conceptual model for designing some components of a serious game to promote self-image and improve health literacy in the field of nutrition and physical activity among adolescents. It links theory, empirical evidence directly provided by the target population, and participatory practice where students act as co-researchers.

Future research is needed to provide a more comprehensive understanding of the model effectiveness and scalability and to identify areas for potential improvement as current results addresses only some aspects of the overall project.

## Figures and Tables

**Table 1:** Overview and meaning of the Octalysis framework Core Drives.

Core Drives	Goals	Motivation	SDT Alignment	Game Strategy
1—Meaning	To be part of a story with higher meaning	Positive	Autonomy Competence	Narrative
2—Development and Accomplishment	Achieving goals, receiving rewards	Extrinsic Positive		Badges
3—Empowerment of creativity and feedback	Creating something, inspiration and positive emotions	Intrinsic Positive	Autonomy Competence	Booster/buffer
4—Ownership and possession	Conquering and protecting something	Extrinsic		Set Collection Exchangeable Points
5—Social influence and relatedness	Connectedness, comparisons, emotional associations	Intrinsic	Connectedness	Group quests
6—Scarcity and impatience	Aspiring to something difficult, to be a strategist	Extrinsic Negative		Loot Box
7—Unpredictability and curiosity	Facing randomness and uncertainty	Intrinsic Negative	Autonomy Competence Connectedness	Mystery Boxes
8—Loss and avoidance	Avoidance behaviour	Negative		Countdown Timers

## Data Availability

The raw data supporting the conclusions of this article will be made available by the authors on request.
